# The Effect of the Extraction Method on the Content of Bioactive Compounds and the Biological Activity of *Nigella sativa* Extracts

**DOI:** 10.3390/molecules30244736

**Published:** 2025-12-11

**Authors:** Romuald Gwiazdowski, Krzysztof Juś, Krzysztof Kubiak, Róża Biegańska-Marecik, Agnieszka Waśkiewicz, Daniela Gwiazdowska

**Affiliations:** 1 Research Centre for Registration of Agrochemicals, Institute of Plant Protection-National Research Institute, Władysława Węgorka 20, 60-318 Poznań, Poland; r.gwiazdowski@iorpib.poznan.pl (R.G.); k.kubiak@iorpib.poznan.pl (K.K.); 2 Department of Natural Science and Quality Assurance, Institute of Quality Science, Poznań University of Economics and Business, Al. Niepodległości 10, 61-875 Poznań, Poland; krzysztof.jus@ue.poznan.pl; 3 Department of Food Technology of Plant Origin, Poznań University of Life Sciences, Wojska Polskiego 31, 60-624 Poznań, Poland; roza.marecik@up.poznan.pl; 4 Department of Chemistry, Poznań University of Life Sciences, Wojska Polskiego 75, 60-625 Poznań, Poland; agat@up.poznan.pl

**Keywords:** food chain safety management, extraction method, biological activity, antimicrobial activity, antioxidant activity

## Abstract

Ensuring food safety and consumer health are crucial elements of sustainable food safety management, requiring the use of substances that inhibit the growth of undesirable microorganisms at various stages of production. The negative impact of many of these on human health and the environment has led to increased interest in alternative solutions, such as plant extracts. The aim of this study was to determine the biological activity of extracts obtained from *Nigella sativa* seeds using various methods, including Soxhlet and ultrasound-assisted extraction (UAE) using hexane and methanol and supercritical CO_2_ extraction (SFE) assisted with methanol. The content of polyphenolic compounds, their composition, and biological activity depended on the extraction method and solvent type. All extracts exhibited antimicrobial activity against Gram-positive bacteria (*Listeria monocytogenes*, *Priestia megaterium*, and *Staphylococcus aureus*), Gram-negative bacteria (*Salmonella* Enteritidis, *Pseudomonas aeruginosa*, and *Escherichia coli*), yeasts (*Candida albicans* and *Rhodotorula mucilaginosa*), and filamentous fungi (*Alternaria brassicicola*, *Pythium* sp., *Fusarium culmorum*, and *F. graminearum*). The MIC values were in the range of <0.125 to 2 mg/mL for bacteria and 1 to 8 mg/mL for fungi, depending on the extract. Microscopic observations performed using optical and fluorescence microscopy showed changes in the viability and morphology of the fungal cells. TPC values ranged from 9.877 mg/g in hexane extract obtained by ultrasound-assisted extraction to 39.064 mg/g in extract obtained by Soxhlet method with methanol. No negative effects of the extracts on wheat seed germination were observed. Analysis of the composition of polyphenolic compounds revealed the presence of vanillic acid, hydroxybenzoic acid, syringic acid, protocatechuic acid, and *p*-coumaric, catechin, and epicatechin acids in all extracts. The extracts obtained with methanol, both by the Soxhlet method and by ultrasound-assisted extraction, also contained gallic acid, myricetin, luteolin, apigenin, and sinapic acid. In all extracts, thymoquinone ranged from 8.344 mg/g to 63.125 mg/g of extract, which was detected with the highest concentration in hexane extracts.

## 1. Introduction

The challenges of projected population growth, climate change, and ongoing environmental degradation, highlighted in recent decades, require a sustainable approach to ensuring food safety and consumer health. Safe food involves limiting the growth of pathogenic microorganisms throughout the food chain, both during field cultivation and food processing. Crops worldwide are attacked by various pathogens, including fungi, bacteria, viruses, nematodes, and protozoa, responsible for plant diseases, which significantly impact agricultural productivity by reducing yields by up to several dozen percent [1]. Fungi pose a significant problem, as they are not only responsible for plant diseases and serious crop losses but also produce mycotoxins that pose a risk to human and animal health. Contamination of food and feed with toxigenic fungi and mycotoxins leads both to health and socio-economic implications [2,3,4]. It is also emphasized that mycotoxin contamination is responsible for the waste of approximately 1.3 billion tons of food per year, which corresponds to one-third of global food production [5]. At the next stages of the food chain, contamination of food systems with pathogenic bacteria such as *Salmonella* sp. and *Listeria monocytogenes* is a serious problem, leading to numerous illnesses and even death. According to WHO estimates, 33 million disability-adjusted life years (DALYs) are lost globally each year due to the consumption of unsafe food [6]. Microbiological contamination may occur during the storage, processing, and preservation of food and negligence in the hygiene of workers and the production environment, but it may also be related to the adaptation of pathogens to new environments, the formation of biofilms, the acquisition of virulence factors, and the development of resistance to the antimicrobial agents used [7,8,9].

Ensuring food safety requires the use of various preventive methods, including antimicrobial substances. In plant cultivation, in addition to the introduction of new disease-resistant plant varieties, the main strategy for combating pathogens is the use of chemical pesticides. Their widespread use affects various ecosystems, because residues of these compounds, often difficult to degrade into non-toxic compounds, enter the environment, accumulate in the tissues of living organisms, and enter food [10,11]. In turn, the use of various preservatives in food processing and preservation may have negative health effects and also leads to the increasing phenomenon of antimicrobial resistance. The application of different types of biocides including pesticides or pharmaceuticals has also led to the selection of resistant microorganisms, which pose a threat to the environment [12,13]. The use of synthetic chemical compounds with antimicrobial and antioxidant properties is met with increasing public disapproval. Moreover, growing consumer demand for organic, preservative-limited, or “clean label” food products is also a significant factor driving the trend of searching for natural alternatives to synthetic preservatives such as plant extracts [14].

*Nigella sativa* L., commonly known as black cumin, is an annual plant belonging to the buttercup family (*Ranunculaceae*), found mainly in western Asia, the Mediterranean and North Sea regions and western and southern Europe [15]. *N. sativa* seeds have attracted considerable scientific interest due to their nutritional and pharmacological potential. They contain a variety of bioactive compounds, significant amounts of protein (20–30%), carbohydrates, unsaturated fatty acids, and essential minerals, including calcium, iron, and potassium [16,17,18,19]. Black cumin also exhibits strong antioxidant properties, associated with a high content of phenolic acids and flavonoids [19,20]. These compounds efficiently neutralize free radicals, enhance the activity of endogenous antioxidant enzymes such as catalase and superoxide dismutase (SOD), and prevent lipid peroxidation in cell membranes [21,22]. The combined antioxidant and antimicrobial activities contribute to the protective and therapeutic effects of black cumin, including hepatoprotective, cardioprotective, and immunomodulatory actions [18,23]. The antimicrobial activity of *N. sativa* extracts and essential oils has been extensively documented against a wide range of pathogens. Inhibitory effects have been observed against both Gram-positive bacteria, such as *Staphylococcus aureus* and *Bacillus subtilis*, and Gram-negative bacteria, including *Escherichia coli* and *Pseudomonas aeruginosa* [18,24,25]. Recent studies further confirm that black cumin extracts demonstrate significant antibacterial and antiparasitic activity, including the inhibition of biofilm formation and bacterial quorum sensing [26].

Various extraction methods are employed to isolate the bioactive compounds from *N. sativa* seeds, depending on the chemical nature of the target compounds and the intended use of the extract. Conventional methods include maceration and Soxhlet extraction, using ethanol, methanol, hexane, or chloroform to obtain fixed oils and polyphenols [18,27]. While effective, these methods are time-consuming and require large volumes of solvents. Modern techniques, such as ultrasound-assisted extraction (UAE) and microwave-assisted extraction (MAE), improve the extraction efficiency while reducing the solvent consumption and processing time [28,29,30]. Supercritical CO_2_ extraction (SFE) has gained popularity as an eco-friendly and efficient technology that allows the production of high-purity extracts. This method utilizes CO_2_ in a supercritical state, often combined with a co-solvent (ethanol or methanol) under controlled temperature and pressure conditions [31].

Considering the above data, the present work aimed to investigate the biological activity and composition of *N. sativa* extracts obtained via different methods including Soxhlet extraction and ultrasound-assisted extraction, as well as supercritical dioxide extraction. The novelty of the presented research is the comprehensive approach to the characterization of *N. sativa* extracts including a broad assessment of the biological activity along with the composition of phenolic compounds. Most authors focused on broad characteristics of a single extract or compared extracts within a single method using different solvents, usually concentrating on the yield and composition, as well as on selected properties, such as the antioxidant activity (in the case of polyphenolic compounds). This study comprehensively compared the extraction methods, solvent types, and composition of the extracts obtained, as well as their broad biological activity. It is also worth emphasizing that there is still a lack of data on the broader characteristics of CO_2_ extracts, especially in relation to extracts obtained using the most popular extraction methods.

## 2. Results

### 2.1. Antimicrobial Activity of N. sativa Extracts

#### 2.1.1. Antibacterial Activity

The antibacterial properties of the tested *N. sativa* extracts were determined against six indicator bacteria including Gram-positive and Gram-negative species. The obtained results showed high activity of all extracts towards all examined bacteria. The higher activity was observed towards Gram-positive bacteria, where the MIC and MBC values were at the level of <0.125 to 1.0 mg/mL (Table 1). The most susceptible bacteria were *P. megaterium*, while the sensitivity of *L. monocytogenes* was generally lower. *S. aureus* was strongly inhibited by extracts obtained by Soxhlet and ultrasound-assisted extraction; however, extract NS-CO_2_ inhibited bacterial growth at a concentration of 1.0 mg/mL. Gram-negative bacteria were less sensitive. The MIC values were 0.5 to 1.0 mg/mL, and the MBC values were 0.5 to 2.0 mg/mL. The lowest susceptibility was demonstrated by *E. coli*. Comparing the activity of individual extracts, it could be stated that the extracts obtained by Soxhlet and ultrasonic-assisted extraction showed similar antibacterial properties, while the activity of the CO_2_ extract was lower; however, it should be emphasized that effect depended on the indicator microorganism.

**Figure 1:**
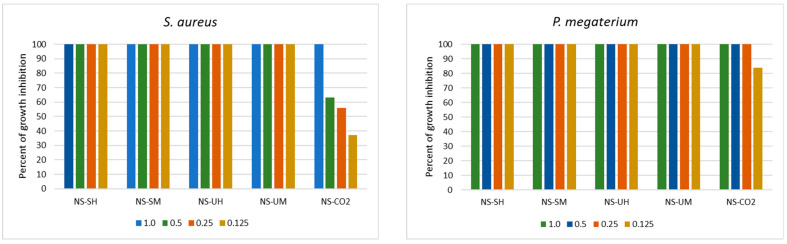

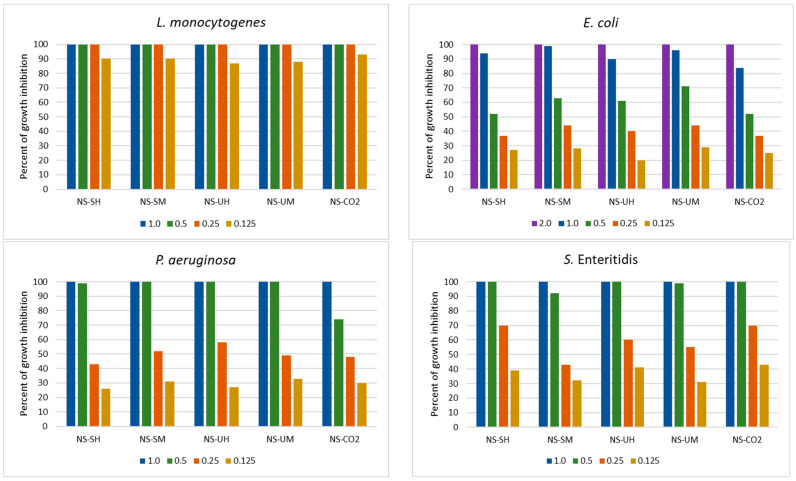
Antibacterial activity of *N. sativa* extracts in the range of concentrations 0.125 to 2.0 mg/mL (NS-SH—*N. sativa* extract obtained by the Soxhlet extraction with hexane; NS-SM—*N. sativa* extract obtained by the Soxhlet extraction with methanol; NS-UH—*N. sativa* extract obtained by the UAE with hexane; NS-UM—*N. sativa* extract obtained by the UAE with methanol; NS-CO_2_—*N. sativa* extract obtained by supercritical CO_2_ extraction).

The MIC value was determined for the extract concentration that inhibited the growth of the indicator microorganism by at least 90%; however, most extracts, even at lower concentrations, showed strong inhibition of the growth of microorganisms (Figure 1).

For example, MIC values for extracts NS-UH and NS-UM were determined at a level 0.25 mg/mL for *L. monocytogenes*; however, a lower concentration of these extracts inhibited bacterial growth in 87–88%. *P. megaterium* was also strongly inhibited (84%) by the lowest used concentration, although the MIC value was established at a concentration of 0.25 mg/mL. In turn, in relation to Gram-negative bacteria, a drastic decrease in growth-inhibiting activity was noted at concentrations below the MIC value. A similar decrease in activity was observed when *S. aureus* was treated with the extract NS-CO_2_.

#### 2.1.2. Antifungal Activity

The conducted research showed that the *N. sativa* extracts also demonstrated antifungal activity towards yeasts and filamentous fungi. The examined yeasts were more susceptible to the effects of extracts with MIC and MFC values in the range 1.0 to 2.0 mg/mL (Table 2) with higher activity towards *R. mucilaginosa*. For filamentous fungi, the MIC and MFC values were higher and reached values 2.0 to 8.0 mg/mL, depending on the fungal strain (Table 2 and Figures S1–S4 in Supplementary Materials). In the case of *A. brassicicola* and *Pythium* spp., the MIC and MFC values were at the same level, while for the remaining fungi, differences in the activity of individual extracts were observed. The highest sensitivity to *N. sativa* extracts was observed for *F. graminearum*, against which MIC and MFC values of 2.0 mg/mL were recorded for the extract obtained by Soxhlet extraction with hexane. In turn, *F. culmorum* was the most susceptible to extracts obtained both Soxhlet and ultrasound-assisted extraction with hexane. Figures S1–S4 visually demonstrate the inhibitory effect of extracts on the filamentous fungi growth.

**Figure 2:**
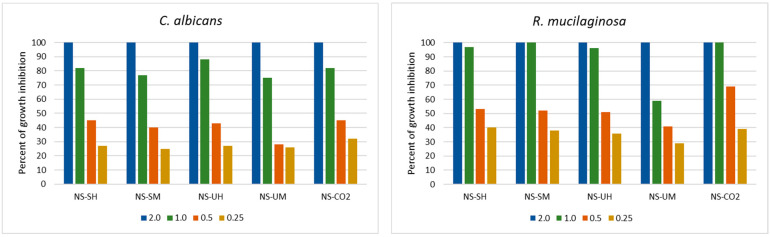
Activity of *N. sativa* extracts towards yeasts in the range of concentrations 0.125 to 2.0 mg/mL (NS-SH—*N. sativa* extract obtained by the Soxhlet extraction with hexane; NS-SM—*N. sativa* extract obtained by the Soxhlet extraction with methanol; NS-UH—*N. sativa* extract obtained by the UAE with hexane; NS-UM—*N. sativa* extract obtained by the UAE with methanol; NS-CO_2_—*N. sativa* extract obtained by supercritical CO_2_ extraction).

In relation to yeasts, a similar strong decrease in the activity of extracts below the MIC value was observed as in the case of Gram-negative bacteria (Figure 2). In addition, the activity of the NS-CO_2_ extract was comparable to the other extracts, and at lower concentrations, it inhibited yeast growth even more strongly than others. This was especially noticeable in the case of *R. mucilaginosa* (69% and 55% of growth inhibition at concentrations 0.5 and 0.25 mg/mL, respectively). The remaining extracts inhibited the growth of these species by 41–53% and 29–40% at concentrations of 0.5 and 0.25 mg/mL, respectively).

**Figure 3:**
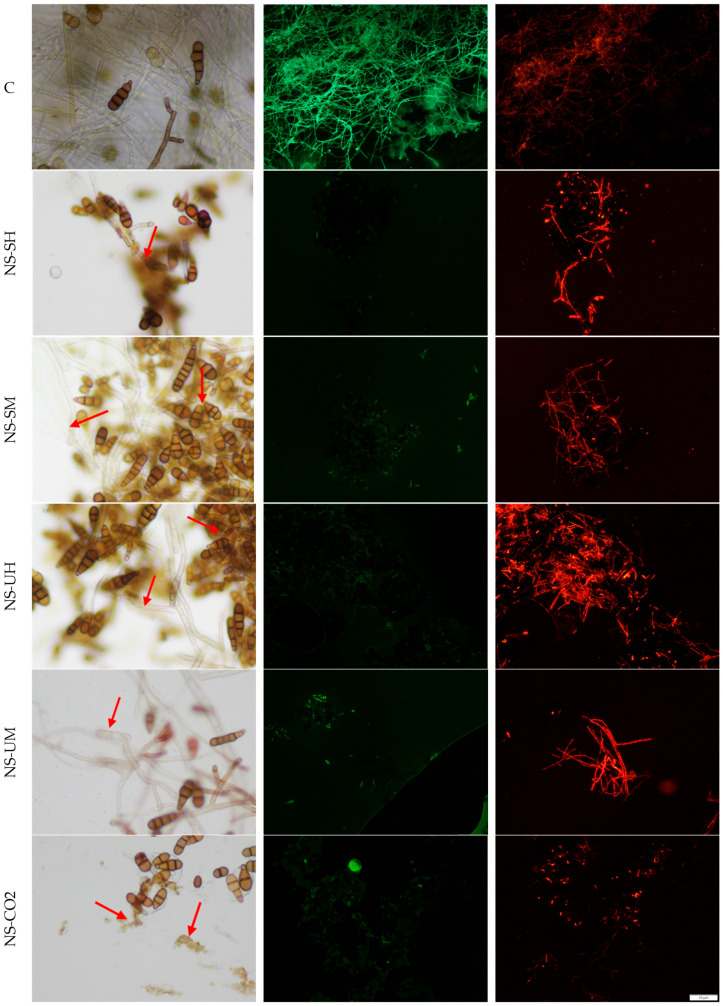
Morphology of *A. brassicicola* hyphae under an optical microscope (magnification 400×), left column, and fluorescence microscope (magnification 100×), columns: middle and right, (C) untreated control, stained with FDA (green) and PI (red), (NS-SH, NS-SM, NS-UH, NS-UM, NS-CO_2_) samples treated with extracts, under an optical microscope from left and stained with FDA (green) and PI (red). The red arrows indicate examples of places where the destructive influence of extracts can be seen.

**Figure 4:**
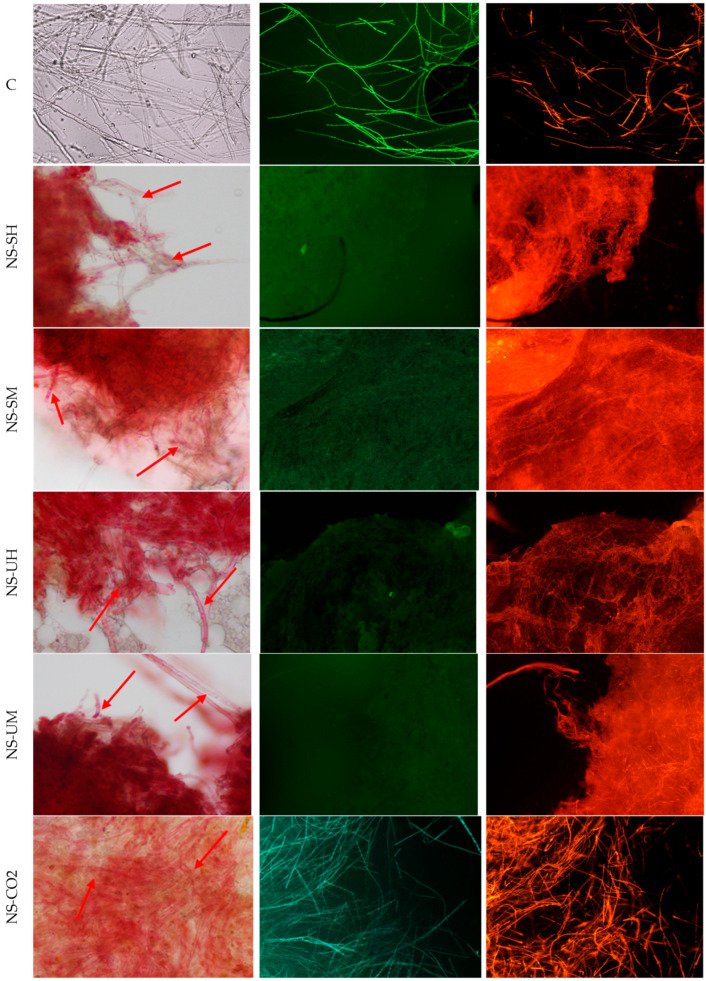
Morphology of *F. graminearum* hyphae under an optical microscope (magnification 400×), left column, and fluorescence microscope (magnification 100×), columns: middle and right, (C) untreated control, stained with FDA (green) and PI (red), (NS-SH, NS-SM, NS-UH, NS-UM, NS-CO_2_) samples treated with extracts, under an optical microscope from left and stained with FDA (green) and PI (red). The red arrows indicate examples of places where the destructive influence of extracts can be seen.

The fungistatic activity of the tested *N. sativa* extracts was also confirmed in microscopy studies by analyzing the effect of the extracts on mature mycelium on the example of two fungal species: *A. brassicicola* and *F. graminearum*. Living cells were detected in the sample treated with NS-CO_2_ extract, which was probably related to the phenomenon of cell aggregation, which could hinder the extract from penetrating into the deeper layers of the mycelium. Microscopic analyses were performed by incubating mycelia with extracts at a concentration of 8 mg/mL for 1 h. The results are presented in Figure 3 and Figure 4.

Based on the obtained microscopic images, it can be concluded that all the tested extracts affect both the structure and viability of hyphae and spores. In the case of *A. brassicicola*, the formation of spore aggregates in samples treated with extracts, as well as the deformation and dehydration of hyphae, was observed. NS-CO_2_ extract caused spore destruction (microscopic images show fragments of spores after their disintegration). Microscopic images of *F. graminearum* showed a disintegration of structures within the fungal hyphae and morphological changes, such as swelling and bending. NS-CO_2_ extract caused the highest morphological changes, resulting in the hyphae clumping into a uniform mass with difficult-to-distinguish individual hyphae. The destructive effect of the extracts is confirmed by fluorescence microscopy images (Figure 3 and Figure 4) obtained after the use of fluorescein diacetate (FDA) and propidium iodide (PI) dyes. A predominance of dead hyphae (red) was observed after incubation of mycelium with the extracts compared to control samples (a higher number of live hyphae—green).

### 2.2. The Effect of N. sativa Extracts on Seed Germination

Determining the effect of the tested extracts on grain germination was important from the perspective of their practical application in biological plant control. In the first round of grain counting, only the NS-SH extract significantly reduced the grain germination (85.3% of seeds germinated) compared to the negative treatment, which did not receive any active agent. In the remaining treatments, the number of germinated seeds ranged from 90.8% to 96.0%, with the lowest value obtained for the PC treatment (seeds treated with 50% ethanol), while the best effect was observed with the CO_2_ extract. After 8 days of incubation, no significant effect of the tested extracts on seed germination was observed compared to the control treatments. The number of germinated seeds exceeded 96%, with the lowest number of seeds regerminating in the presence of the NS-SH extract (Table 3).

### 2.3. Characterization of Phenolic Composition and Antioxidant Properties of N. sativa Extracts

The extracts were analyzed for their phenolic composition (Table 4). The extraction efficiency, depending on the method and solvent (Table 5), ranged from 15.30% (NS-SM) to 26.85% (NS-CO_2_). The extraction efficiency of the Soxhlet and ultrasound-assisted extraction were comparable considering the solvent used and was 16.10–16.30% for hexane and 15.30–15.50% for methanol.

Compound contents in the extracts were expressed as the combined amounts of the parent compounds and their derivatives. For example, this included p-hydroxybenzoic acid together with its esters, catechin with catechin gallate, and p-coumaric acid with its ester forms; the same approach was applied to the remaining compounds.

The composition of phenolic compounds and antioxidant activity was differentiated depending on the extraction method and solvent (Table 4). The highest concentration of phenolic compounds at the level of 37.564–39.064 mg/g of extract (NS-UM and NS-SM, respectively) was found in methanol extracts obtained both by the Soxhlet extraction method and the ultrasound-assisted extraction method. The composition of the extracts was similar, and the dominant compounds were syringic acid and protocatechuic acid. Both extracts contained a comparable amount of hydroxybenzoic acid, catechin acid, epicatechin, gallic acid, and vanillic acid. The extract NS-UM was the only one that contained sinapic acid. In turn, the amount of myricetin and luteolin were higher than in the extract NS-SM, while the concentration of *p*-coumaric acid was higher in extract NS-SM. A high amount of total phenolic acid (29.220 mg/g) was also determined in the extract, obtained by supercritical CO_2_ extraction. Fewer compounds were identified in this extract, and the dominant compounds in this extract were hydroxybenzoic acid and syringic acid. It is worth noting that the content of these two components was much higher than in the other extracts. The extracts obtained with hexane contained the fewest phenolic compounds with hydroxybenzoic acid as the dominant compound. The content of polyphenolic compounds corresponded to the antioxidant activity of the extracts. The highest activity was demonstrated in extracts NS-UM and NS-SM, while the lowest antioxidant activity was in extracts obtained with hexane as solution.

In all extracts, the presence of thymoquinone was found, the primary bioactive compound of *N. sativa*, the content of which ranged from 8.344 mg/g to 63.125 mg/g of extract, with the highest concentration recorded in the extracts obtained with hexane. In turn, the lowest concentration was recorded in extracts obtained by supercritical CO_2_ extraction.

## 3. Discussion

Food safety is a global issue with a significant impact on human health and requires a comprehensive approach, based on the “farm to fork” concept, which assumes a sustainable approach at every stage of food production [32]. The concept of sustainable development plays a significant role in agriculture and food production, given that ensuring safe food starts in the field, ends with the finished meal, and includes economic, social and environmental aspects [33,34]. Controlling the growth and transmission of pathogenic microorganisms in the food chain involves the use of various chemicals, such as fungicides that inhibit the growth of pathogenic fungi in the field or preservatives or antibiotics in the food industry. However, their mass use increases the risk of microorganisms becoming resistant and having a negative impact on human health as well as the environment and its biodiversity [35,36,37]. Therefore, the demand for alternative antimicrobial agents is growing, and black cumin has gained significant research attention due to its high content of a wide range of bioactive compounds such as terpenes and terpenoids including thymoquinone as the most important bioactive compound due to its various therapeutic effects on the human body, alkaloids, fatty acids or phytosterols [38,39,40,41]. Among the various bioactive compounds in *N. sativa* seeds, one important component is polyphenols, which influence antioxidant properties and support antimicrobial activity. Phenolic compounds are naturally occurring substances found in plants, in leaves, stems, roots, flowers, and fruits [42]. They are widely reported to have antioxidant and anti-inflammatory properties that may help prevent chronic diseases, but their importance as natural preservatives is also emphasized, with various functions, such as protection against ultraviolet radiation, pathogens, and insects [43,44]. Studies indicate the high antimicrobial potential of these compounds. They have been shown to have antibacterial properties against foodborne pathogens, including *Escherichia coli*, *Salmonella* spp., *Clostridium perfringens*, *Staphylococcus aureus*, and *Listeria monocytogenes* [45,46,47,48]. The literature data also indicate that these compounds may act synergistically with clinical antimicrobials, increasing the effectiveness of antimicrobials while limiting the development of antimicrobial resistance in foodborne pathogens [49]. In addition, antifungal properties of phenolic compounds are reported, mainly towards pathogenic yeasts such as *Candida* species [50,51].

In the presented study, all extracts demonstrated antibacterial and antifungal activity against a wide spectrum of microorganisms. The antibacterial activity was stronger towards Gram-positive bacteria with *P. megaterium* as the most susceptible indicator strain. In turn, among Gram-negative bacteria, the most sensitive microorganism was *S.* Enteritidis, and the least sensitive was *E. coli*. Similarly, Saleh et al. [52] observed a stronger inhibitory effect of methanolic extracts (undiluted, diluted with water 1:1) against Gram-positive strains than against Gram-negative strains. Shafodino et al. [53] reported the antibacterial activity of different extracts of *N. sativa* seeds against Gram-negative (*E. coli* and *P. aeruginosa*) and Gram-positive (*S. aureus* and *B. subtilis*) bacteria in the disc diffusion method. Other authors also confirm that phenolic compounds exhibit higher activity against Gram-positive strains compared to Gram-negative strains, suggesting it is due to the presence of an outer membrane in the cell wall of Gram-negative bacteria, which acts as a permeability barrier and limits the absorption of polyphenols [54,55]. However, not all authors observed a similar relationship. For example, in the study by Zouirech et al. [56], the antimicrobial effect of essential oil from *N. sativa* obtained by hydrodistillation was evaluated against four clinically important bacterial strains with stronger activity against *E. coli* K12 and *Bacillus subtilis* DSM 6333 than against *S. aureus*, ATCC 6633, and *Proteus mirabilis* ATCC 29906). The reason for such differences may be the extraction method and the composition of the extracts. The obtained extracts also demonstrated antifungal properties towards the yeasts *C. albicans* and *R. mucilaginosa* and filamentous fungi belonging to *Fusarium* species, as well as *A. brassicicola* and *Pythium* sp. Our results are in line with the literature data indicating the antifungal activity of *N. sativa* bioactive compounds against fungi including *Candida albicans* [57,58], *P. digitatum*, *C. gloeosporioides* [59], *Trichophyton* sp., *Microsporum canis* [60], *Aspergillus niger*, *A. flavus*, *Alternaria* [61], *Candida tropicalis*, *Trichophyton mentagrophytes*, *Epidermophyton floccosum*, and *Penicillium* [62].

Comparing the biological properties of individual extracts is difficult due to the variety of extraction methods, extraction conditions, and solvents used by the authors. This also applies to polyphenolic compounds. Muzolf-Panek and Gliszczyńska-Świgło [63] emphasize that the polyphenol profile results from the extraction procedure. Different authors describe various extraction techniques such as microwave-assisted extraction, ultrasound-assisted extraction, supercritical fluid extraction, enzyme-assisted extraction, pulsed electric field extraction, or accelerated solvent extraction [64,65,66,67]. In the presented work, the TPC values differed between extracts, ranging from 9.877 mg/g in the hexane extract obtained by ultrasound-assisted extraction to 39.064 mg/g in the extract obtained by the Soxhlet method with methanol. The literature data confirm the high differences between various extraction methods. In the study of Dar et al. [26], the total phenolic content (TPC) of the *N. sativa* seed oil extracted by the three methods revealed that the TPC of oil extracted by ultrasound assisted extraction was higher (214.17 ± 0.24 mg GAE/100 mL) than both the conventional Soxhlet extraction (CSE) with n-hexane and supercritical carbon dioxide extraction (SCE) methods (211.32 ± 0.36 and 208.78 ± 0.28 mg GAE/100 mL respectively). In this study, the antioxidant properties corresponded to the content of polyphenolic compounds with the highest antioxidant activity of oil extracted by the ultrasound-assisted extraction (UAE) method and higher than that of the CSE and SCE methods. Similar observations have been made in the presented work. The phenolics content was comparable in the extracts obtained by Soxhlet and ultrasound-assisted extraction and higher than in the extract obtained by supercritical fluid extraction, while the antioxidant activity was the highest for the extract obtained by the ultrasound-assisted extraction.

Furthermore, various authors have observed that the TPC level during the extraction of polyphenolic compounds is significantly influenced by the solvent concentration, time, and extraction conditions. For example, in the study of Soleimanifar et al. [68], the TPC value was 0.96 mg GAE/g in extracts obtained using 80% aqueous methanol, conducting the extraction for 28 h in ambient temperature, while Hameed et al. reported 2.93 mg GAE/g for 70% methanol extract [69]. Dar et al. [26] observed that the extraction temperature, extraction time, and solvent concentration had a significant effect on the yield and antioxidant activity of the extracted *N. sativa* oil.

The type and conditions of extraction also influence the composition of polyphenolic compounds in the extracts. In this work, the composition of phenolic compounds and antioxidant activity was differentiated depending on the extraction method and solvent, which is consistent with the literature data [70,71]. In all of the prepared extracts, vanillic acid, hydroxybenzoic acid, syringic acid, protocatechuic acid, and *p*-coumaric, catechin and epicatechin acid were detected. The extracts obtained with methanol, both by the Soxhlet method and by ultrasound-assisted extraction, also contained gallic acid, myricetin, luteolin, apigenin, and sinapic acid (only in NS-UM) and had the richest composition. In all extracts, the presence of thymoquinone, a monoterpenoid compound that is a primary biologically active ingredient was also detected. The individual identified components influence the biological properties of the extracts. For example, caffeic acid and catechins have strong antioxidant properties, while vanillic acid is known for its neuroprotective activity [72,73,74]. It is worth noting that the extracts demonstrated the strongest antioxidant activity in the present work contained the highest concentration of caffeic acid, while the extracts NS-SM and NS-UM also contained the highest amount of catechins. Moreover, thymoquinone, despite the lack of any phenol hydroxyl group associated with antioxidant activity, shows antioxidant properties, described by many authors [75,76,77], which also explains the obtained results.

The literature data describe the antibacterial and antifungal activity of phenolics; however, the activity spectrum and mechanism of action depend on the structure and properties of phenolic compounds. The antibacterial activity is usually more widely described in the literature, while the antifungal properties are less well-known. For example, gallic acid, detected in the extracts NS-SM and NS-UM, is a bioactive compound that is a natural component of many traditional Chinese medicines and is widely considered safe for humans [78]. Its antibacterial, antiviral, antifungal, anti-inflammatory, and antioxidant effects have been demonstrated, leading to growing interest in its use in the food industry and medicine [79,80]. In Tian et al.’s study [47], GA demonstrated antibacterial activity against clinical isolates of MDR *Escherichia coli* as well as the inhibition of bacterial biofilm formation. Interestingly, GA enhanced the activity of ceftiofur sodium or tetracycline against *E. coli* and facilitated antibiotic accumulation in bacteria. Syringic acid was reported as an anti-biofilm agent against three biofilm-forming methicillin-resistance *Staphylococcus epidermidis* strains [81]. Stojkovic et al. [82] reported the antibacterial activity of protocatechuic acid against *S. aureus*, *B. cereus*, *M. flavus*, *P. aeruginosa*, *E. coli*, and *Enterobacter cloacae*. This compound is better known for its antibacterial activity, but recent studies indicate that PCA can increase the antimicrobial activity of routinely used antibiotics and antifungal drugs by up to 50% [83]. Xu et al. [84] have found that 4-coumaric acid and p-hydroxybenzoic acid inhibited *C. gloeosporioides* growth with MIC value at a level 1 g/L and 2 g/L, respectively. In the study of Liu et al. [85], p-coumaric acid demonstrated significant antifungal properties towards *B. cinerea* and *Penicillium expansum*. The authors described the inhibition of mycelium and reduction in the patulin production by *P. expansum*. Caffeic acid, syringic acid, and 4-hydroxybenzoic acid (4-HBA) were reported to have high antifungal activity against Ganoderma [86]. Nagar et al. [87] showed the antifungal activity of gallic acid, pyrogallic acid, and syringic acid against *A. solani*, which depended on the applied dose. At the highest concentration (100 ppm), syringic acid showed the strongest effect. In turn, Jaiswal and Kumar [88] showed the strong antifungal potential of chlorogenic acid, caffeic acid, coumaric acid, and 2,4-dihydroxybenzoic acid, with caffeic acid being the most potent antifungal agent. Ponts et al. [89] investigated the effect of five phenolic acids on fungal growth and type B trichothecene production by four strains of *F. graminearum* and reported that the most toxic compound was ferulic acid, followed by p-coumaric acid, syringic acid, caffeic acid, and p-hydroxybenzoic acid. Except for ferulic acid, all the others were present in the extracts tested in this study, which proves that they could have a significant contribution to antifungal activity. Moreover, it is important to note that, depending on the extract, different concentrations of individual compounds were observed, which due to synergistic interaction may enhance or weaken the antimicrobial effect. For example, in the NS-CO_2_ extract, the sum of hydroxybenzoic acid and syringic acid was 27.188 mg/g extract, while in the remaining extracts it did not exceed 7 mg/g extract, which could have resulted in a different mode of action on fungal cells and the stronger destruction of fungal spores, visible in fluorescence microscopy. The antimicrobial effect of the tested extracts was probably also due to the presence of thymoquinone, whose antibacterial and antifungal activity has been widely described. This compound demonstrated antimicrobial activity, for example, against *Listeria monocytogenes*, *Escherichia coli*, *Pseudomonas aeruginosa*, *Staphylococcus aureus*, and *Yersinia enterocolitica* [90,91,92].

The literature data indicate that the mechanism of the antimicrobial activity of phenolic acids is connected with the increase in the cell membrane permeability and the leakage of cell constituents [93,94]. According to their explanation, an undissolved form of phenols passes through the cell membrane and disrupts its structure. The effect may be the acidification of the cytoplasm and protein denaturation. The authors underlined that the differentiated effects of phenolic acids on membrane permeability could depend on the differences in their structure and lipophilic character. Campos et al. [94], in their experiment with lactic acid bacteria, revealed that hydroxycinnamic acids (*p*-coumaric, caffeic, and ferulic acids) induce higher ion leakages and higher proton influx than hydroxybenzoic acids (*p*-hydroxibenzoic, protocatechuic, gallic, vanillic, and syringic acids). This may explain the strongest antibacterial activity of methanolic extracts in this study, considering the fact that they contained higher amounts of hydroxycinnamic acid derivatives.

It is worth emphasizing that various authors have described different compositions of extracts. Muzolf-Panek and Gliszczyńska identified three phenolic compounds in *N. sativa* extracts, including quercetin and kaempferol derivatives in methanolic extracts [63]. Toma et al. [95] and Zwolan et al. [96] noted the presence of kaempferol derivatives as the most abundant phenolic compounds in a 70% ethanolic extract of *N. sativa*, while other authors identified gallic, protocatechuic, p-hydroxybenzoic, chlorogenic, and caffeic acids similarly to the presented work, as well as rutin, quercetin, apigenin, naringenin, and diosmin [69,71,97]. The presence of thymoquinone, recognized as the most important bioactive compound isolated from *N. sativa* seed oil [98] was also described. Many authors indicate the content of thymoquinone in extracts may differ as a result of various factors of extraction, which is in line with the present study. In the study of Iqbal et al. [99], the thymoquinone content was 1.55-fold high in the hexane extract than in the methanol extract, while in the benzene extract, the authors noted 10.0-fold more thymoquinone compared to the methanol extract. A similar relationship was noted in this study.

It is also worth noting that the tested extracts did not affect plant germination, which is important, due to their potential use as an alternative to chemical compounds. Only a short-term germination-limiting effect was observed for the NS-SH extract. The use of plant extracts in agriculture requires that these compounds be environmentally friendly but also non-toxic to plants.

## 4. Materials and Methods

### 4.1. Plant Material

Dried black cumin seeds *Nigella sativa* were purchased from a Polish manufacturer, Naturalnie Zdrowe Sp. z o. o., Wiązowna, Poland. The product had a manufacturer’s quality certificate confirming that it meets certain quality standards and requirements, as well as information about its nutritional value. Before extraction, dried black cumin seeds were ground in an A 11 basic laboratory grinder from IKA Poland Sp. z o. o., Warsaw, Poland.

### 4.2. Nigella Sativa Sample Extraction

#### 4.2.1. Extraction Methods

The methods used to prepare the extracts were Soxhlet, ultrasound, and supercritical CO_2_ extraction, using different solvents. Soxhlet extraction was performed based on the modified method by Karami et al. [100]. The extract was obtained from 10 g of ground black cumin seeds weighed into extraction vessels in a Soxhlet apparatus using 250 mL of two different solvents: methanol and hexane.

Ultrasound-assisted extraction was performed in an Elmasoic P ultrasonic bath (Elma Schmidbauer GmbH, Singen, Germany), based on the methodology described by Hossain et al. [101], with some modifications. Ten grams of ground black cumin seeds were weighed into 200 mL flasks, and 50 mL of methanol or hexane were added. The samples were placed in an ultrasonic bath and extraction was performed in three 15 min cycles at a frequency of 50 kHz and 35 °C. After each cycle, another 50 mL portion of solvent was added, yielding a final 150 mL extraction mixture. All extracts were then filtered on a Buchner funnel under increased pressure.

The extraction was carried out using the supercritical CO_2_ method in accordance with the procedure described by Uwineza et al. [102]. Ground and dried *N. sativa* seeds (5 g) were placed in a 25 mL extraction vessel. The process was conducted at a temperature of 50 °C and a pressure of 250 bar. The CO_2_ flow rate was set to 4 mL/min, while methanol (99.5% purity) was used as a co-solvent at a rate of 1 mL/min. Once the desired operating conditions were achieved, the extraction process was initiated automatically and continued for 180 min for each variant. Each extraction cycle consisted of a first dynamic phase lasting 45 min, a static phase of 15 min, and a second dynamic phase of 120 min. The obtained *N. sativa* extracts were collected in flasks placed within a fraction collection module and stored at −20 °C until further analysis.

#### 4.2.2. Preparation of Extracts for Assays

Before determining the exact mass of the black cumin seed extracts, the extraction solvents were evaporated using a rotary evaporator. The mass of the extract obtained was then calculated based on the weight before and after evaporation. Based on the obtained results, the appropriate amount of solvent required to prepare solutions with a concentration of 32 mg/mL was calculated. The extracts were dissolved in ethanol. The types of extracts, along with the method of obtaining them, the symbols used in this work, and the extract and solvent concentrations, are listed in Table 5.

### 4.3. Chemicals

Methanol was purchased from POCh (Gliwice, Poland). The acetonitrile was purchased from Witko (Łódź, Poland). Carbon dioxide (CO_2_, SFE grade) was purchased from Air Products Sp, Siewierz, Poland. Folin–Ciocalteu’s reagent and hydrochloric acid of 35–38% purity were purchased from Chempur (Piekary Śląskie, Poland). Gallic acid, chlorogenic acid, quercetin, sodium acetate, 2,2′-azino-bis(3-ethylbenzothiazoline-6-sulfonic acid (ABTS), and (±)-6-hydroxy-2,5,7,8-tetramethylchromane-2-carboxylic acid (Trolox) were purchased from Sigma-Aldrich (Merck Life Science sp. z o.o., Poznań, Poland). Microbiological media were purchased from BioMaxima (Lublin, Poland) and A&A Biotechnology (Gdańsk, Poland). All chemicals were of analytical grade.

### 4.4. Determination of Antimicrobial Activity of N. sativa Extracts

#### 4.4.1. Indicator Microorganisms

The antibacterial activity of the tested extracts was determined according to Gwiazdowska et al. [103] towards three Gram-positive bacteria—*Staphylococcus aureus* ATCC 33862, *Priestia megaterium* WG006, and *Listeria monocytogenes* ATCC 19115), as well as three Gram-negative bacteria—*Escherichia coli* ATCC 8739, *Pseudomonas aeruginosa* ATCC 9027, and *Salmonella enterica* ser. Enteritidis ATCC 13076. The antifungal activity was determined against two strains of yeast—*C. albicans* ATCC 10231 and *Rodotorula mucilaginosa* DKK 050, as well as four filamentous fungi of the genus *Fusarium* (*F. graminearum* KZF 1 and *F. culmorum* KZF 5), *Pythium* sp. KZF 50, and *Alternaria brassicicola* BPR 1735. The indicator strains were purchased from the American Type Culture Collection (ATCC, Manassas, VA, USA), the collection of the Department of Natural Science and Quality Assurance (DKK, WG), the Research Centre for Registration of Agrochemicals (KZJ), and the Bank of Plant Pathogens and Research on their Biodiversity (BPR), Institute of Plant Protection National Research Institute in Poznań, Poland.

#### 4.4.2. Inoculum Preparation and Standardization

Bacteria and yeasts were cultured for 24 h on agar media: nutrient agar (NA) for *S. aureus*, *P. megaterium*, *E. coli*, and *P. aeruginosa*, brain heart infusion agar (BHI) for *L. monocytogenes*, and *S.* Enteritidis and yeasts on Sabouraud Dextrose Agar (SAB), under 37 °C. Next, inocula in Mueller–Hinton broth (MHB) for bacteria and SAB Broth with an optical density adjusted to 0.5 McFarland standard were prepared.

The tested filamentous fungi were cultivated in Petri dishes (55 mm diameter) on a Potato Dextrose Agar (PDA) at 25 °C for 5–10 days. Subsequently, hyphae and conidia suspensions were prepared in sterile PDB by mixing harvested mycelium from mature cultures with medium to achieve a final cell concentration of 10^6^ cells/mL, determined with a hemocytometer [103].

#### 4.4.3. Minimal Inhibitory Concentration (MIC), Minimal Bactericidal Concentration (MBC), and Minimal Fungicidal Concentration (MFC) Determination

The MIC, as well as the MBC/MFC, of the tested *N. sativa* extracts were determined using the microdilution method according to Gwiazdowska et al. [103]. Twofold dilutions of the extracts were prepared in 96-well microtiter plates in MHB for bacteria, SAB for yeast, and PDB for filamentous fungi. The final concentration of the tested extracts was established in the range of 0.125–16 mg/mL. Appropriate culture media containing *N. sativa* extracts without microorganisms were used as negative controls, whereas bacterial or fungal cultures without extracts were used as positive controls. The final concentration of ethanol in the control samples was established in the range of 0.4–50% in proportion to its content in the samples. Next, 100 µL of the microorganism solutions prepared according to Section 4.4.2. were added to each well. The plates inoculated with bacteria and yeasts were covered and incubated for 24 h at 30 °C or 37 °C, depending on the microorganism. In the case of filamentous fungi, microtiter plates were sealed with parafilm (to minimize the risk of extracts evaporation) and incubated at 25 °C ± 2 °C for 5–10 days under aerobic conditions. After incubation, the optical density of the bacterial and yeasts samples was determined at a 600 nm wavelength using the BioTek Epoch 2 microplate reader (Winooski, VT, USA). The MIC value was defined as the lowest concentration of extract that exhibited at least 90% growth inhibition, and the MBC value was determined as 100% inhibition. The MFC value was determined via spot inoculation of 10 μL of microbial culture. After incubation, observations were made to determine at which concentrations the growth was inhibited. The lowest concentration that completely inhibited fungal growth was taken as the MFC value (Figures S1–S4 in Supplementary Materials). All tests were performed in triplicate.

### 4.5. Phenolic Composition

The phenolic profile was determined by HPLC in an LC Agilent Technologies 1200 Rapid Resolution system equipped with a UV–Vis detector (DAD 1260) (Agilent Technologies 1200 Rapid Resolution, Waldbronn, Germany) and a Zorbax SB-C18 column, with grain size of 5 μm and dimensions of 4.6 × 150 mm (Agilent Technology Inc., Santa Clara, CA, USA). Separation was performed in a reverse phase system using gradient elution. The mobile phase was 60 g/L acetic acid in 2 mmol sodium acetate solution (solvent A) and 100% acetonitrile (solvent B) [104]. The system was run with a gradient program: 0–15% A for 15 min, 15–30% B for 10 min, 30–50% B for 5 min, and 50–100% B for 5 min, pre-run to the initial composition for 5 min. The flow rate was 1 mL/min, and the total run time was 40 min. Compounds were identified based on spectral analysis and supported by the literature data. Spectral similarity (DAD) was assessed using the Agilent OpenLab CDS ChemStation C.01.10(201) “spectral match” factor, calculated from the correlation between the absorbance values at each wavelength (match factor = r^2^ × 1000). Based on the maximum wavelength of UV–Vis absorption and the retention time, the compounds were quantified at 250, 280, 320 nm, and 360 nm using the external standard method. Thymoquinone, gallic acid, caffeic acid, and quercetin were used as standards for the compounds detected at the respective wavelengths. Concentrations of individual compounds found in each sample were determined using the calibration curves generated from standards. The results were expressed in µmol/g of extract. Standard deviations for the means ranged from 0.5 to 8%.

### 4.6. Determination of Antioxidant Activity (TEAC) of N. sativa Extracts

The radical scavenging capacity of *N. sativa* extracts was assessed by the TEAC (Trolox Equivalent Antioxidant Capacity) assay [105]. The samples of extracts were centrifuged in a MiniSpin centrifuge (Eppendorf) (Eppendorf Poland Sp.z o.o, Warsaw, Poland) for 5 min at 10,000 rpm and diluted to a concentration of 10 mg/mL. The diluted samples were mixed with ABTS•+ solution, and after 6 min of incubation in the dark (ambient temperature), the decrease in the absorbance was measured in a Genesys 10S UV–VIS spectrophotometer (Thermo Fisher Scientific, Waltham, MA, USA). TEAC values were expressed in μmol Trolox equivalent per g of extract. TEAC values were calculated as the equation of the sample dilution ratio and the corresponding Trolox standard curve.

### 4.7. The Effect of N. sativa Extracts on Wheat Seed Germination

The effect of *N. sativa* extracts on wheat seed germination was determined according to the International Seed Testing Association (ISTA) standard [106] using the Arkadia variety. The extracts were added to 50 g of wheat grain in the amount of 0.5 mL and then shaken in a tight plastic container for 1 min. The same application method was used for the positive control, where 0.5 mL of 50% ethanol was added. The negative control consisted of grain without any treatment. After drying, control samples (without extracts) and samples spiked with extracts were placed on glass plates lined with damp filter paper and incubated under appropriate conditions (20 °C, dark) for 8 days to allow germination. The first count of germinated seeds was made after 4 days and the next after 8 days. Each plate contained 100 seeds, and each test item was replicated four times.

### 4.8. Light and Fluorescence Microscopy

*A. brassicicola* and *F. graminearum* were cultured in PDA (Potato Dextrose Agar) for 5 days at 25 °C. After incubation, the mycelia of the tested fungi were removed from the surface of the medium and suspended in 1 mL of sterile water with the addition of *N. sativa* extracts at a concentration equal to 1 × MIC for 1 h and 2 h of incubation. Samples were transferred on glass slides, observed, and photographed with a light microscope (Olympus BX53, Olympus Corporation, Tokyo, Japan) at 400× magnification. Fungal samples treated with a mixture water: ethanol (1:1) were used as control samples. Each assay was repeated in triplicate.

The viability of *A. brassicicola* and *F. graminearum* treated with *N. sativa* extracts was assessed by staining with the fluorescent probes, fluorescein diacetate (FDA), and propidium iodide (PI) (Sigma Aldrich, Steinheim, Germany). The staining procedure was performed according to the method described by Wang et al., 2022 [107], and Gwiazdowska et al., 2022 [108]. First, a stock solution of FDA (1 mg/mL) in acetone and a stock solution of PI (1 mg/mL) in distilled water were prepared and stored in the refrigerator. After treatment of the fungal strains with *N. sativa* extracts for 1 h, the samples were centrifugated, and 1 mL of sterile water was added to the mycelia. Next, 25 μL FDA and 5 μL PI were added to the samples, which were incubated with fluorescent dyes for 30 min at 30 °C. The staining solution was removed, and the pellet was transferred onto glass slides and examined under the fluorescence microscope Olympus BX53 (Olympus Corporation, Tokyo, Japan), equipped with specific wavelength filters set (FDA excitation/emission: 485/530; PI excitation/emission: 538/617). Fungal samples treated with water: ethanol (1:1) were used as control samples.

### 4.9. Statistical Analysis

The results are presented as the arithmetic mean (±standard deviation) from three parallel replicates. In addition, selected results (wheat seed germination and antioxidant activity) were estimated by one-way analysis of variance (ANOVA) using Tukey’s test with a significance level of *p* < 0.05. For all statistical analyses, the Microsoft Excel^®^ and IBM SPSS Statistics 29 (PS IMAGO PRO 9.0) programs were used.

## 5. Conclusions

In this study, Soxhlet, ultrasound-assisted, and supercritical fluid extraction were used to obtain extracts and compare their biological activity, taking into account potential application possibilities. The obtained extracts differed in their phenolic content and composition. The methanolic extracts obtained with both Soxhlet and ultrasound-assisted extraction were characterized by the most diverse composition and the highest phenolics content, which could have contributed to their strong antimicrobial properties. The CO_2_ extract, on the other hand, was characterized by a very high content of acid derivatives, which could contribute to its strong destructive effect on fungal spores. The results demonstrated the activity of the tested extracts against a broad spectrum of microorganisms, both bacteria and fungi. Despite differences in the composition and content of polyphenolic compounds, the antimicrobial activity of the tested extracts was quite similar, as all extracts inhibited all tested indicator microorganisms within a similar concentration range. However, differences in antioxidant activity were observed, related to the different content of polyphenolic compounds. Methanol extracts were characterized by both a higher content of polyphenolic compounds and higher antioxidant activity. Among the extracts obtained with the use of methanol, the SC-CO_2_ extract showed lower polyphenol content and antioxidant activity. However, it is of particular interest due to the fact that the SFE process involves the use of ecologically clean technology, offering the potential for inclusion.

## Figures and Tables

**Table 1:** Antibacterial activity (MIC/MBC) of *N. sativa* extracts against bacteria.

Indicator Bacteria	MIC and MBC of *N. sativa* Extracts * [mg/mL]					
		NS-SH	NS-SM	NS-UH	NS-UM	NS-CO_2_
Gram-positive bacteria						
*S. aureus*	MIC	<0.125	<0.125	<0.125	<0.125	1
	MBC	<0.125	<0.125	<0.125	<0.125	1
*P. megaterium*	MIC	<0.125	<0.125	<0.125	<0.125	0.125
	MBC	<0.125	<0.125	<0.125	<0.125	0.25
*L. monocytogenes*	MIC	0.125	0.125	0.25	0.25	0.125
	MBC	0.25	0.25	0.25	0.25	0.25
Gram-negative bacteria						
*E. coli*	MIC	1	1	1	1	1
	MBC	2	2	2	2	2
*P. aeruginosa*	MIC	0.5	0.5	0.5	0.5	1
	MBC	1	0.5	0.5	0.5	1
*S. enteritidis*	MIC	0.5	0.5	0.5	0.5	0.5
	MBC	0.5	1	0.5	0.5	0.5

* NS-SH—*N. sativa* extract obtained by the Soxhlet extraction with hexane; NS-SM—*N. sativa* extract obtained by the Soxhlet extraction with methanol; NS-UH—*N. sativa* extract obtained by the UAE with hexane; NS-UM—*N. sativa* extract obtained by the UAE with methanol; NS-CO_2_—*N. sativa* extract obtained by supercritical CO_2_ extraction.

**Table 2:** Antifungal activity (MIC/MFC) of *N. sativa* extracts against yeasts and filamentous fungi.

Indicator Fungi	MIC and MFC of *N. sativa* Extracts * [mg/mL]					
		NS-SH	NS-SM	NS-UH	NS-UM	NS-CO_2_
Yeasts						
*C. albicans*	MIC	2	2	2	2	2
	MFC	2	2	2	2	2
*R. mucilaginosa*	MIC	1	1	1	2	1
	MFC	2	1	2	2	1
Filamentous fungi						
*A. brassicicola*	MIC	4	4	4	4	4
	MFC	4	4	4	4	4
*F. culmorum*	MIC	4	8	4	8	8
	MFC	4	8	4	8	8
*F. graminearum*	MIC	2	4	4	4	4
	MFC	2	4	4	4	4
*Pythium* spp.	MIC	8	8	8	8	8
	MFC	8	8	8	8	8

* NS-SH—*N. sativa* extract obtained by the Soxhlet extraction with hexane; NS-SM—*N. sativa* extract obtained by the Soxhlet extraction with methanol; NS-UH—*N. sativa* extract obtained by the UAE with hexane; NS-UM—*N. sativa* extract obtained by the UAE with methanol; NS-CO_2_—*N. sativa* extract obtained by supercritical CO_2_ extraction.

**Table 3:** Effect of the tested extracts on grain germinability under laboratory conditions.

*N. sativa* Extracts	Grain Germinability [%]	
	After 4 Days	After 8 Days
NS-SH	85.3 ^a^ ± 2.1	96.0 ^a^ ± 1.4
NS-SM	91.3 ^ab^ ± 2.4	98.3 ^a^ ± 2.9
NS-UH	93.0 ^b^ ± 1.4	98.5 ^a^ ± 0.6
NS-UM	93.8 ^b^ ± 3.5	98.3 ^a^ ± 1.7
NS-CO_2_	96.0 ^b^ ± 0.8	98.3 ^a^ ± 1.5
PC	90.8 ^ab^ ± 3.8	99.0 ^a^ ± 0.8
NC	93.5 ^b^ ± 3.9	97.5 ^a^ ±1.7

NS-SH—*N. sativa* extract obtained by the Soxhlet extraction with hexane; NS-SM—*N. sativa* extract obtained by the Soxhlet extraction with methanol; NS-UH—*N. sativa* extract obtained by the UAE with hexane; NS-UM—*N. sativa* extract obtained by the UAE with methanol; NS-CO_2_—*N. sativa* extract obtained by supercritical CO_2_ extraction; PC—positive control; NC—negative control; averages with different letters (^a,b^) are significantly different at *p* ˂ 0.05 based on one-way analysis of variance (ANOVA) using Tukey’s test.

**Table 4:** Content of thymoquinone and phenolic compounds in mg/g of extract and antioxidant activity expressed as μmol/g.

*N. sativa* Extracts	Derivatives of Hydroxybenzoic Acid				Derivatives of Hydroxycinnamic Acid			Flavanols			Flavonols			NI	TPC ***	TQ	Antioxidant Activity μmol/g ****
	GA	VA *	HA *	SA *	PTA *	P-CA *	CFA *	SP *	CA **	ECA *	MI	LT	AP				
NS-SH	-	0.072	3.313	0.375	0.938	0.344	-	-	1.250	0.938	-	-	-	0.14	11.605	60.687	21.63 ^b^ ± 1.18
NS-SM	1.125	0.438	2.781	4.125	5.938	2.344	8.438	-	2.656	1.406	5.000	0.188	0.375	0.136	39.064	47.313	44.91 ^c^ ± 0.97
NS-UH	-	0.063	2.813	0.313	0.156	0.250	-	-	1.375	0.844	-	-	-	0.13	9.877	63.125	21.00 ^b^ ± 1.15
NS-UM	1.094	0.438	2.781	4.156	5.938	1.125	0.469	5.625	2.656	1.406	6.688	0.625	0.313	0.136	37.564	34.696	52.19 ^d^ ± 1.19
NS-CO_2_	-	0.313	18.125	9.063	0.125	0.125	0.250	-	1.094	0.125	-	-	-	-	29.220	8.344	10.17 ^a^ ± 0.71

* Presented as the sum of the compounds and their derivatives, ** presented as the sum of catechin and catechin gallate, *** TPC—total phenolic compound—does not include TQ, **** antioxidant activity (TEAC value) was expressed in μmol Trolox equivalent per g of extract, NS-SH—*N. sativa* extract obtained by the Soxhlet extraction with hexane; NS-SM—*N. sativa* extract obtained by the Soxhlet extraction with methanol; NS-UH—*N. sativa* extract obtained by the UAE with hexane; NS-UM—*N. sativa* extract obtained by the UAE with methanol; NS-CO_2_—*N. sativa* extract obtained by supercritical CO_2_ extraction, TQ—thymoquinone, GA—gallic acid, VA—vanillic acid, HA—hydroxybenzoic acid, SA—syringic acid, PTA—protocatechuic acid, P-CA—*p*-coumaric acid, CFA—caffeic acid, SP—sinapic acid, CA—catechin, ECA—epicatechin, MI—myricetin, LT—luteolin, AP—apigenin, NI—not identified; for antioxidant activity, averages with different letters (^a–d^) are significantly different at *p* ˂ 0.05, based on one-way analysis of variance (ANOVA) using Tukey’s test.

**Table 5:** Characteristics of *N. sativa* extracts.

Extract	Extraction Solvent	Yield of Extraction (%)	Dissolving Solvent	Extract Concentration (mg/mL)
Soxhlet extraction				
NS-SH	hexane	16.10	ethanol	32
NS-SM	methanol	15.30	ethanol	32
ultrasound-assisted extraction				
NS-UH	hexane	16.30	ethanol	32
NS-UM	methanol	15.50	ethanol	32
supercritical CO_2_ extraction				
NS-CO_2_	methanol	26.85	ethanol	32

## Data Availability

The data presented in this study are available in the article and in the Supplementary Materials.

## Supplementary Materials

The following supporting information can be downloaded at: https://www.mdpi.com/article/10.3390/molecules30244736/s1, Figure S1. Antifungal activity of *N. sativa* extracts against *A. brassicicola*, Figure S2. Antifungal activity of *N. sativa* extracts against *F. culmorum*, Figure S3. Antifungal activity of *N. sativa* extracts against *F. graminearum*, Figure S4. Antifungal activity of *N. sativa* extracts against *Pythium* spp.
