# Supplementary material for: The Effect of the Extraction Method on the Content of Bioactive Compounds and the Biological Activity of Nigella sativa Extracts
Source: Molecules. 2025 Dec 11;30(24):4736. doi: 10.3390/molecules30244736 (PMC12736113; doi:10.3390/molecules30244736)
Supplement: Supplementary file 1 [file molecules-30-04736-s001.zip › molecules-3999244-supplementary.pdf]

molecules

MDPI

Supplementary Materials

The Effect Of The Extraction Method On The Content Of Bioactive Compounds And The Biological Activity Of *Nigella Sativa* Extracts

Romuald Gwiazdowski [1], Krzysztof Juś [2], Krzysztof Kubiak [1], Róża Biegańska-Marecik [3], Agnieszka Waśkiewicz [4] and Daniela Gwiazdowska [2,*]

[1] Research Centre for Registration of Agrochemicals, Institute of Plant Protection-National Research Institute, Władysława Węgorka 20, 60-318 Poznań, Poland; r.gwiazdowski@iorpib.poznan.pl (R.G.), (K.K.)
[2] Department of Natural Science and Quality Assurance, Institute of Quality Science, Poznań University of Economics and Business; krzysztof.jus@ue.poznan.pl (K.J.)
[3] Department of Food Technology of Plant Origin, Poznan University of Life Sciences, Poznań, Poland; roza.marecik@up.poznan.pl (R.B.M.)
[4] Department of Chemistry, Poznań University of Life Sciences, Wojska Polskiego 75, 60-625 Poznań, Poland; agat@up.poznan.pl (A.W.)
* Correspondence: daniela.gwiazdowska@ue.poznan.pl (D.G.)

1. Supplementary Materials

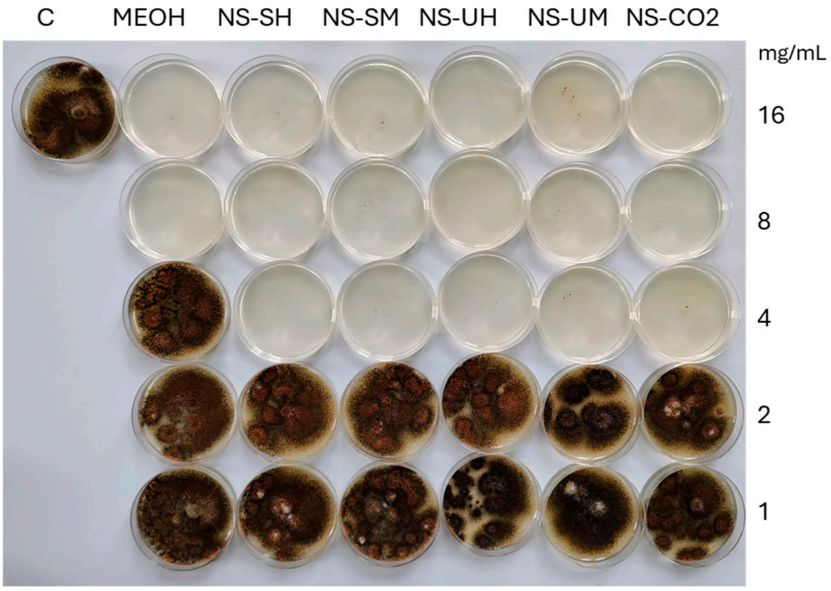

Figure S1. Antifungal activity of *N. sativa* extracts against *A. brassicicola*

 https://doi.org/10.3390/xxxxx

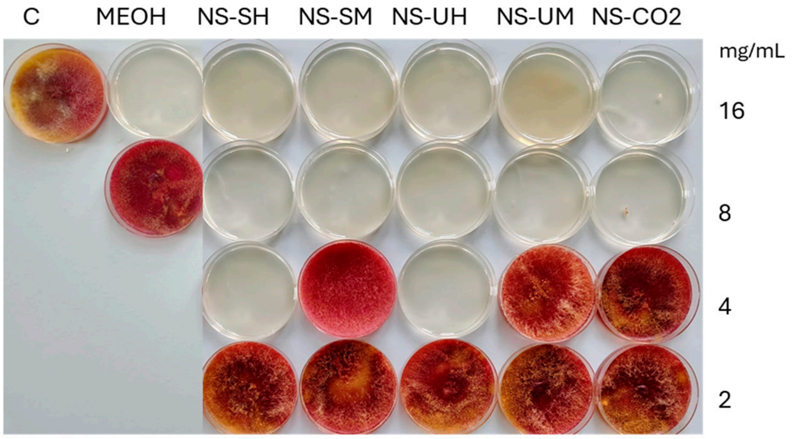

Figure S2. Antifungal activity of *N. sativa* extracts against *F. culmorum*

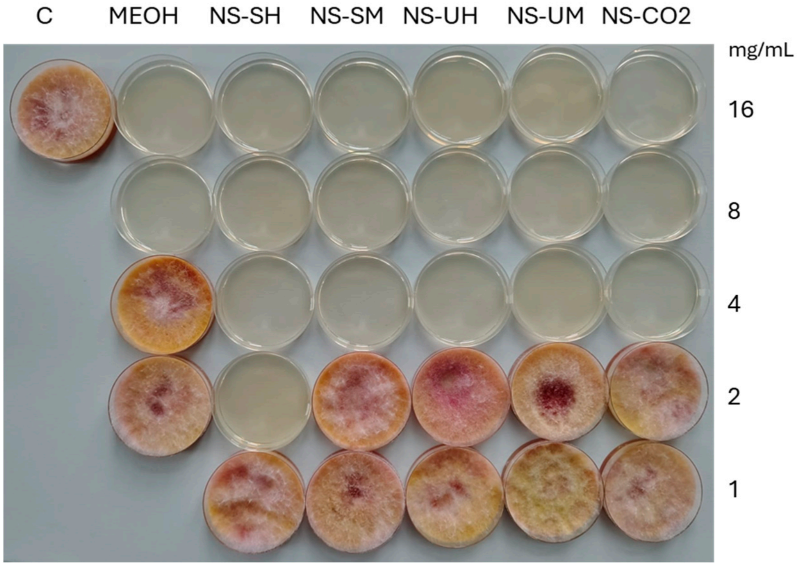

Figure S3. Antifungal activity of *N. sativa* extracts against *F. graminearum*

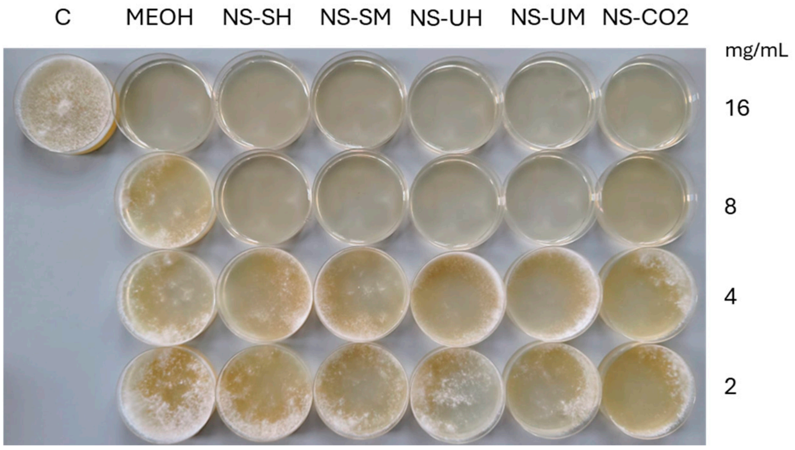

Figure S4. Antifungal activity of *N. sativa* extracts against *Pythium spp.*
